# Genome-Wide Identification and Expression Analysis of the *HCT* Gene Family in Upland Cotton (*Gossypium hirsutum* L.) in Response to *Verticillium wilt* Infection

**DOI:** 10.3390/biology15070520

**Published:** 2026-03-25

**Authors:** Yujia Zhang, Gang Liu, Baojun Liu, Mengxue Zhang, Yang Hu, Shu Wang, Jidi Sun, Aixing Gu

**Affiliations:** 1 College of Agriculture, Xinjiang Agricultural University, Urumqi 830052, China; zyj970226@163.com (Y.Z.); liu09997776@163.com (G.L.); baojunliulky@163.com (B.L.); mengxuezhang67233@163.com (M.Z.); hy3159585094@163.com (Y.H.); w572391676@126.com (S.W.); sunjidi424313@163.com (J.S.); 2 Engineering Research Centre of Cotton, Ministry of Education, Urumqi 830052, China; 3 Institute of Economic Forestry, Xinjiang Academy of Forestry Sciences, Urumqi 830052, China

**Keywords:** *Gossypium hirsutum*, *Verticillium dahliae* L., *HCT* gene family, expression analysis

## Abstract

Cotton, a globally vital cash crop, is severely constrained by *V. dahliae*. Lignin, a core structural component of plant cell walls, plays a crucial role in physical defense, with its biosynthesis regulated by hydroxycinnamoyltransferase (HCT)—a key enzyme in the phenylpropanoid pathway. However, the *HCT* gene family in upland cotton (*Gossypium hirsutum*) and its role in resistance to *V. dahliae* remain poorly understood. In this study, we performed a genome-wide identification of the *HCT* gene family in *G. hirsutum*, identifying 74 *GhHCT* genes that were classified into five evolutionary subfamilies. Bioinformatics analysis revealed that GhHCT proteins exhibit conserved functional domains but diverse gene structures, with promoter regions enriched in hormone-responsive and stress-responsive cis-acting elements. Expression profiling revealed that multiple *GhHCT* genes were significantly induced in response to *V. dahliae* infection. Three genes, *GhHCT2*, *GhHCT35*, and *GhHCT47*, showed significantly higher expression levels in resistant cultivars than in susceptible cultivars during early infection stages, suggesting pivotal roles in defense. These three candidate genes, which contain MeJA/SA-responsive elements in their promoters, may enhance resistance by regulating lignin synthesis to strengthen the cell wall barrier. In summary, this study provides the first comprehensive characterization of the *HCT* gene family in upland cotton. It identifies key candidates for improving resistance to *V. dahliae*, offering valuable genetic resources for molecular breeding.

## Simple Summary

Cotton production is severely threatened by *Verticillium wilt*, a disease caused by the fungus *Verticillium dahliae* L. This study identified 74 *HCT* genes in the cotton genome and found that three—*GhHCT2*, *GhHCT35*, and *GhHCT47*—are rapidly and strongly activated in resistant plants upon infection, suggesting that they may serve as key defense candidates by regulating lignin biosynthesis. These findings provide valuable genetic resources for breeding cotton varieties with enhanced resistance to this devastating disease.

## 1. Introduction

Cotton (*Gossypium* spp.) ranks among the most crucial cash crops globally, with its fibers occupying a central position in the global textile industry chain and playing a crucial role in both global and Chinese economic development [1,2]. However, throughout its entire growth cycle, cotton is severely constrained by *V. dahliae*. This pathogen produces highly resilient sclerotia that can survive in soil for over a decade without losing pathogenicity [3,4,5]. Owing to the lack of effective chemical control methods and the commercial availability of highly resistant varieties, *V. dahliae* is extremely difficult to manage, leading the industry to be nicknamed “cotton cancer” [2,5,6,7]. Consequently, unraveling the mechanisms of cotton disease resistance and identifying key resistance genes have emerged as critical scientific challenges in cotton molecular breeding.

Over time, plants have developed multilayered defense systems to counter pathogen invasion, with strengthened cell walls and physical barriers serving as key strategies for disease resistance [8,9]. Recent studies have indicated that lignin, the primary phenolic polymer in secondary cell walls, not only enhances the mechanical strength of cell walls to effectively prevent pathogen invasion and spread but also plays a central role in systemic plant defense responses [2,10]. From a molecular structural perspective, lignin is a complex reticular polymer formed by the polymerization of three cinnamyl alcohol monomers, and its deposition level directly affects cell wall permeability and mechanical properties [11]. Of particular note, during infection of cotton by *V. dahliae*, the rapid deposition impacts multiple biotic stresses, including pathogen infections, insect feeding, and weed competition, which significantly impair yield and fiber quality [2,12,13]. *Verticillium wilt*, caused by *V. dahliae*, ranks among the most severe diseases limiting cotton production [2,14,15]. Lignin in infected vessel walls acts synergistically with tylosis formation to establish a dual physical barrier that restricts the vascular spread of the pathogen [16,17,18].

Lignin biosynthesis relies on the highly conserved phenylpropanoid pathway, where hydroxycinnamoyl-CoA shikimate is the key rate-limiting enzyme in this pathway, whose catalytic activity directly determines the composition ratio and deposition pattern of lignin monomers [2,19,20,21,22]. Genomic studies have revealed that HCTs typically form multigene families in plants. The functional diversity among family members is achieved through structural variation, differential expression patterns, and specific protein interactions, characteristics that are of significant importance in plant secondary metabolic regulatory networks [22,23,24]. Recent functional studies indicate that HCTs not only participate in lignin biosynthesis but also play regulatory roles in plant responses to various abiotic stresses, such as nitrogen stress, low temperatures, and drought [2,24,25]. However, notably, in the allopolyploid *G. hirsutum*, the scale and characteristics of the *HCT* gene family, as well as the mechanisms underlying their functional differentiation under *V. dahliae* stress, remain poorly understood. In particular, the potential functional division of labor and synergistic regulatory networks among *HCT* genes originating from different subgenomes (At and Dt) have not been systematically elucidated.

Despite the established importance of *HCT* genes in lignin biosynthesis and stress responses across plant species, a systematic characterization of this gene family in allotetraploid *G. hirsutum*—particularly in the context of defense against the devastating pathogen *V. dahliae*—has been lacking. Therefore, this study conducted a genome-wide identification and systematic analysis of the *HCT* gene family in *G. hirsutum*, focusing on the following key tasks: comprehensively elucidating the physicochemical properties of its encoded proteins, their phylogenetic relationships, gene structures, and chromosomal distribution characteristics; investigating the expansion and differentiation patterns of *HCT* genes in tetraploid cotton through comparative genomics; and specifically utilizing transcriptome data to analyze the expression dynamics of this gene family in detail during *V. dahliae* infection. This study aimed to identify key *GhHCT* genes involved in cotton resistance to *V. dahliae*, providing new insights into the molecular mechanisms of cotton disease resistance and offering important candidate genes for genetic improvement in disease resistance in cotton.

## 2. Materials and Methods

### 2.1. Plant Materials and Treatments

The cotton materials used in this experiment were the hybrid lines disease-resistant material (10Q-11-2) and susceptible material (10Q-67) [26], which were preserved at the College of Agriculture, Xinjiang Agricultural University. The plants were cultivated in a greenhouse (23–28 °C, 16 h light/8 h dark). When the cotton plants reached the two-leaf-and-one-bud stage, we inoculated with a suspension of *V. dahliae* (10^7^ CFU/mL, 20 mL per pot) using the root-cutting and dipping method [27]. Under identical cultivation conditions, samples were collected at 0 h, 3 h, 6 h, 9 h, 12 h, 24 h, and 48 h post-inoculation with *V. dahlia* (the *V. dahliae* strain used in this study was Vd991, provided by the Plant Pathology Laboratory of Xinjiang Agricultural University). The sampling sites are leaves, with three replicates per sample. The 0 h time point for each sample serves as the control group. Fresh samples were rapidly frozen in liquid nitrogen and stored at −80 °C in ultralow-temperature freezers. Additionally, at approximately 25 days post-inoculation, photographs were taken to document plant growth and disease symptoms (Supplementary Figure S1).

### 2.2. Identification of GhHCT Genes

The *Arabidopsis thaliana* HCT protein sequence (AT5G48930) was obtained from the TAIR database (https://www.arabidopsis.org/, accessed on 1 December 2025). *G. hirsutum* genome data, protein sequence data, and GFF3 annotation information (TM-1-T2T) were downloaded from CottonGen (https://www.CottonGen.org/, accessed on 6 December 2025) [28]. The *A. Thaliana* HCT protein sequence was used as the query, and BLASTP (2.17.0) was run with default parameters to search the *G. hirsutum* protein database (e-value < 0.001). Structural prediction was subsequently performed on the sequences obtained in the previous step using the Pfam database (http://pfam.xfam.org/, accessed on 6 December 2025) and the SMART website (http://smart.embl-heidelberg.de/, accessed on 8 December 2025). Sequences lacking the typical HCT protein domain were excluded, and the remaining protein sequences were considered to be members of the *GhHCT* family.

### 2.3. Physicochemical Properties and Chromosomal Localization Analysis of Proteins

ExPaSy (https://web.expasy.org/protparam/, accessed on 10 December 2025) was used to analyze the amino acid composition, average total hydrophilicity, isoelectric point, and molecular weight of GhHCT. WoLF PSORT (https://wolfpsort.hgc.jp/, accessed on 10 December 2025) was employed to predict the subcellular localization of the GhHCT protein. The chromosomal locations of all *GhHCT* genes in the *G. hirsutum* genome were extracted and visualized using Tbtools (v2.441) [29].

### 2.4. Gene Structure and Conserved Motif Analysis

The *GhHCT* gene structure information was downloaded from the *G. hirsutum* genome database (https://cottonfgd.org/, accessed on 10 December 2025). A phylogenetic tree was constructed from sequences using MEGA7.0 and saved in nwk format. Motif prediction was performed using MEME (http://meme-suite.org/tools/meme, accessed on 10 December 2025), set to search for 10 motifs, saved in XML format, and finally visualized using TBtools.

### 2.5. Analysis of Promoter Cis-Acting Elements

The Gff3 Sequence Extractor in TBtools was used to extract the 2000 bp promoter region upstream of the *GhHCT* gene’s start codon ATG. This sequence was subsequently submitted to the online promoter analysis tool Plant CARE (http://bioinformatics.psb.ugent.be/webtools/plantcare/html/, accessed on 23 December 2025) for cis-acting element analysis [28]. The predicted elements were curated and visualized via TBtools’ Simple BioSequence Viewer.

### 2.6. Phylogenetic Classification Analysis

A phylogenetic tree was constructed using the neighbor-joining (NJ) method in MEGA 7.0, including five species: 225 HCT protein sequences from five species: 74 from *G. hirsutum*, 1 from *A. thaliana*, 50 from *Nicotiana tabacum*, 50 from *Theobroma cacao*, and 50 from *Oryza sativa*. The number of bootstrap runs was set to 1000, while the other parameters used default settings. The resulting unrooted tree was submitted to ITOL (http://itol.embl.de/, accessed on 26 December 2025) to generate a circular phylogenetic tree.

### 2.7. Collinearity Analysis of the GhHCT Gene Family

To analyze collinearity among *G. hirsutum*, the model plant *A. thaliana*, and tobacco, an interspecies collinearity analysis was performed. The tobacco genome sequence and associated annotation files were downloaded from the IMP website (https://www.bic.ac.cn/IMP/, accessed on 14 December 2025). TBtools was used to visualize the collinearity relationships among *A. thaliana*, *G. hirsutum*, and tobacco, as well as collinearity within *G. hirsutum*.

### 2.8. Expression Analysis of the GhHCT Gene Family

Transcriptome sequencing data from *G. hirsutum* were utilized to obtain *GhHCT* gene expression levels at different time points during *V. dahliae* infection (for details on the sequencing methods, please refer to Supplementary Document S1). Heatmaps were generated via TBtools.

RNA was extracted from samples using the RNA Prep Pure Plant Plus Kit (TIANGEN BIOTECH, Co., Ltd., Beijing, China). Sample quality was assessed via agarose gel electrophoresis and spectrophotometric analysis. cDNA was obtained for each sample using the All-in-One First-Strand Synthesis MasterMix Reverse Transcription Kit (Yugong Biotech Co., Ltd., Lianyungang, Jiangsu, China) for subsequent gene expression analysis [30]. Primer Premier 5.0 was used to design specific primers (Supplementary Table S1). Cotton *GthUBQ7* served as the internal reference gene for qRT-PCR analysis. Relative gene expression levels were calculated using the 2^−ΔΔCt^ method, as detailed in reference [31]. Each dataset originated from 3 biological replicates and 3 technical replicates. The qRT-PCR reaction system comprised 1 μL of template cDNA, 0.2 μL of forward primer, 0.2 μL of reverse primer, 5 μL of SYBRPrimix ExTaqTM (2×), and ddH_2_O to a final volume of 10 μL. qRT-PCR was performed on an ABI 7500 (Applied Biosystems, San Francisco, CA, USA) instrument under the following conditions: 95 °C pre-denaturation for 30 s; 95 °C denaturation for 10 s; 60 °C annealing for 30 s; repeated for 40 cycles.

### 2.9. Prediction of the GhHCT Protein Interaction Network

To investigate HCT protein interactions, we used *A. thaliana* as the reference organism and uploaded the *G. hirsutum* HCT protein sequences to the STRING database (https://cn.string-db.org/, accessed on 15 December 2025). To investigate the protein–protein interactions within the *GhHCT* family, predictions of intra-family interactions were performed following the method of Yang et al. [32]. An interaction score threshold of 0.7 (high confidence) was applied. The results were visualized via Cytoscape 3.10.4 software [2,33].

## 3. Results

### 3.1. Identification of the GhHCT Gene Family and Analysis of Protein Physicochemical Properties

A total of 74 *GhHCT* genes were identified in the *G. hirsutum* genome. Additionally, the amino acid length, theoretical molecular weight, isoelectric point, instability coefficient, fat coefficient, hydrophobicity index, and subcellular localization of GhHCT were analyzed (Table 1). The results indicate that GhHCT proteins comprise 433–498 amino acid residues, with theoretical molecular weights ranging from 47 to 55 kDa and isoelectric points between 5.25 and 9.60. Half of the GhHCT proteins presented instability coefficients of less than 40. With the exception of GhHCT15, all other GhHCT proteins have lipid coefficients of less than 100, indicating that they are lipophilic. Except for nine GhHCT proteins (GhHCT15, GhHCT36, GhHCT37, GhHCT38, GhHCT66, GhHCT71, GhHCT72, GhHCT73, and GhHCT74), the hydrophobicity index (GRAVY) of the remaining GhHCT proteins was less than 0, indicating that they are hydrophilic proteins. Subcellular localization predictions revealed that 44 GhHCT proteins are localized to the cytoplasm, 14 to chloroplasts, 11 to the nucleus, 3 to mitochondria, and 2 to the cytoskeleton (Table 1).

### 3.2. Chromosomal Localization Analysis of the GhHCT Gene Family

**Figure 1:**
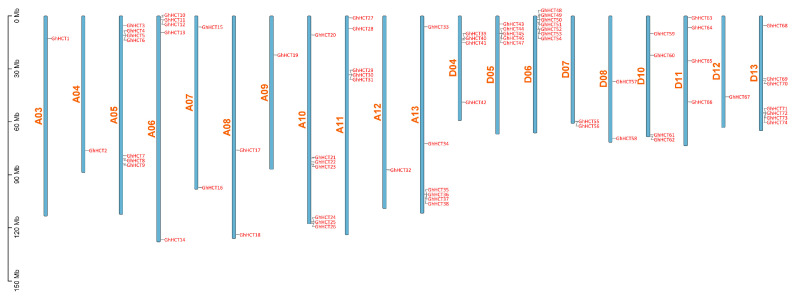
Chromosomal localization analysis of the *GhHCT* gene family. The scale on the left denotes chromosome length. Gene names are mentioned in red. *GhHCT* genes are distributed on 20 chromosomes of *G. hirsutum*.

Members of the *GhHCT* family are distributed across 20 chromosomes of *G. hirsutum* and are unevenly distributed (Figure 1). Chromosomes A05, A10, D06, and D13 each harbor seven *GhHCT* genes. Chromosome A13 contains six *GhHCT* genes, whereas chromosomes A06, A11, and D05 each carry five *GhHCT* genes. Chromosomes D04, D10, and D11 each harbor four *GhHCT* genes, whereas chromosomes A07, A08, D07, and D08 each contain two *GhHCT* genes. Only one *GhHCT* gene is present on chromosomes A03, A04, A09, A12, and D12. Additionally, the *GhHCT* gene exhibited distinct tandem duplication patterns, forming ten tandem gene clusters on chromosomes. The largest tandem cluster contains four genes and spans chromosomes A13, D05, and D13.

### 3.3. Analysis of Conserved Protein Domains and the Gene Structure of the GhHCT Gene Family

**Figure 2:**
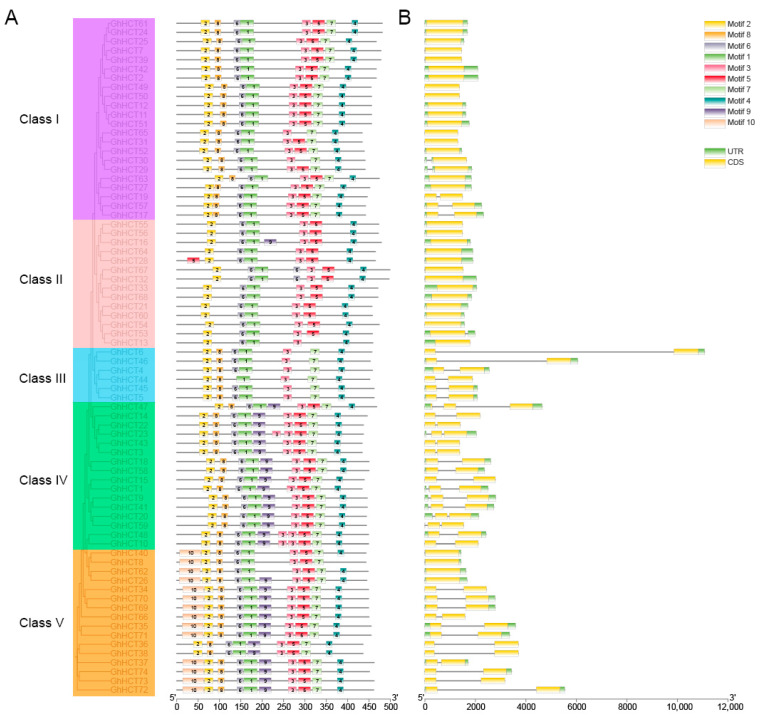
Analysis of the conserved motifs and gene structure of the *GhHCT* gene family. (**A**) *GhHCT* conservative domain analysis. According to the similarity of gene conserved motifs, they are divided into five class, which are represented by different colors. (**B**) Exon–intron structure analysis.

To further investigate the similarities and diversities of motifs among different GhHCT proteins, protein domain prediction was performed using the MEME (5.5.9) software. The results indicate that GhHCT protein structures are relatively conserved, with 10 motifs identified (designated Motifs 1–10). Among these, Motif 1, Motif 2, and Motif 3 presented the highest degree of conservation and were shared across all the subgroups (Figure 2A). In addition to these conserved motifs, each subgroup possessed specific motifs, such as Motif 10, which was unique to the Class V subgroup, and Motif 9, which was present only in the Class IV and Class V subgroups. These motif differences may correlate with the functional diversity of *HCT* genes.

To clarify the *GhHCT* gene structure, the CDSs of each *GhHCT* gene were obtained from the *G. hirsutum* genome, and exon–intron structure diagrams were constructed (Figure 2B). These results indicate that most *GhHCT* family members lack introns. Specifically, 30 members presented two exons and one intron; one member (*GhHCT47*) presented three exons and two introns; six members (*GhHCT1*, *GhHCT9*, *GhHCT20*, *GhHCT23*, *GhHCT41*, *GhHCT59*) presented two exons and two introns; and three members (*GhHCT29*, *GhHCT30*, *GhHCT53*) presented two exons and two introns, while another presented one exon and one intron, indicating diverse gene structures among *GhHCT* family members.

### 3.4. Analysis of cis-Acting Elements in GhHCT Promoters

**Figure 3:**
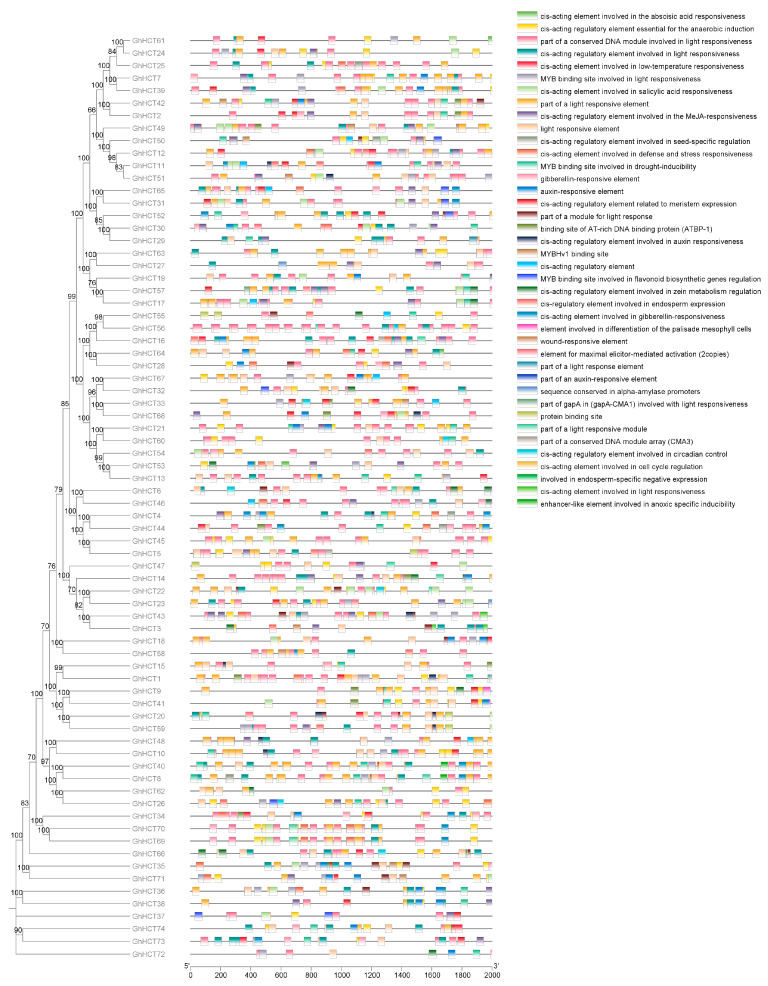
The cis-acting elements of the promoter regions (2000 bp upstream of start codon) of *GhHCT* genes.

The core regulatory function of gene promoters is primarily governed by cis-acting elements located upstream of the transcription start site [2,33,34]. A total of 40 cis-acting elements were identified within the promoter regions, which were categorized into three major functional groups: growth and development, hormone response, and stress response (Figure 3). The stress response elements included anaerobic, cold, drought, and defense/stress responses. Hormone response elements are composed primarily of gibberellin, auxin, salicylic acid, and methyl jasmonate response elements. The presence of these cis-acting elements suggests potential roles for *GhHCT* genes in regulating plant growth and development, hormone responses, and stress responses, although experimental validation is required to confirm these predicted functions.

### 3.5. Phylogenetic Analysis of GhHCT Family Members

**Figure 4:**
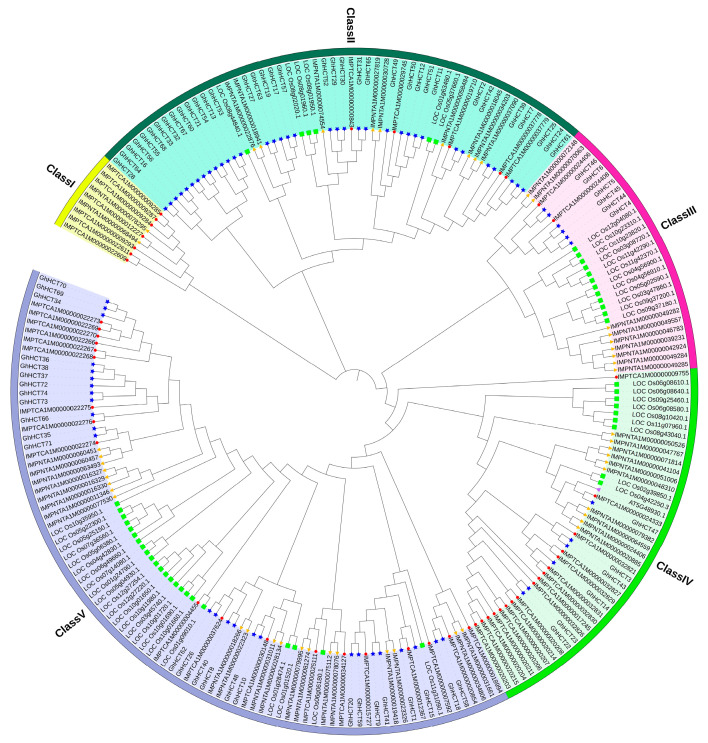
Phylogenetic analysis of the *GhHCT* gene family. The *HCT* genes are divided into Class I, Class II, Class III, Class IV, and Class V, which are represented by different colors. The *HCT* genes of *Theobroma cacao* are represented by red circles, the *HCT* genes of *Nicotiana tabacum* are represented by orange triangles, the *HCT* genes of *G. hirsutum* are represented by blue stars, and the *HCT* genes of *Oryza sativa* and *Arabidopsis thaliana* are represented by green squares and purple triangles.

Phylogenetic analysis grouped the 225 HCTs into five subfamilies (Class I–V) (Figure 4). The Class I subfamily comprises nine HCT members, including two from tobacco and seven from cocoa; the Class II subfamily contains 56 HCT members: 9 from tobacco, 6 from rice, 5 from cocoa, and 36 from *G. hirsutum*; the Class III subfamily comprises 29 HCT members: 9 from tobacco, 12 from rice, 2 from cocoa, and 6 from *G. hirsutum*; the Class IV subfamily comprises 43 HCT members, including 10 from tobacco, 1 from *A. thaliana*, 9 from rice, 17 from cocoa, and 6 from *G. hirsutum*; and the Class V subfamily comprises 88 HCT members, including 20 from tobacco, 23 from rice, 19 from cocoa, and 26 from *G. hirsutum*.

### 3.6. Synteny Analysis of the GhHCT Gene Family

**Figure 5:**
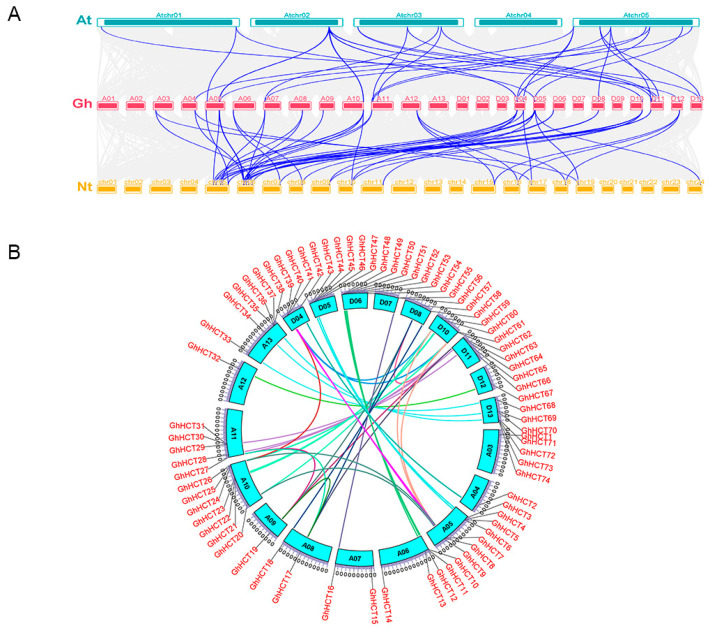
Collinearity analysis of the *GhHCT* family genes. (**A**) Collinearity analysis of *G. hirsutum* with *Arabidopsis thaliana* and *Nicotiana tabacum*. Chromosomes of different species represented by colored bars; gray lines indicate all collinearity modules, while blue lines specifically highlight 76 collinear gene pairs. (**B**) Intraspecific collinearity analysis of the *GhHCT* genes.

To further investigate the homology relationships among *HCT* genes, interspecies synteny analyses between *G. hirsutum* and *A. thaliana*, as well as between *G. hirsutum* and tobacco, were performed at the whole-genome level to explore the evolutionary origins of the *G. hirsutum HCT* gene family (Figure 5A). The analysis revealed 27 and 49 collinear gene pairs between the *GhHCT*, *A. thaliana,* and tobacco genomes, respectively, indicating homologous relationships among *HCT* family genes in *G. hirsutum*, *A. thaliana*, and tobacco. These homologous gene pairs may share similar functions.

Intraspecific synteny analysis within *G. hirsutum* revealed 35 syntenic relationships (Figure 5B). Chromosome A05 harbored the greatest number of homologous gene pairs (*GhHCT3* with *GhHCT43*; *GhHCT7* with *GhHCT24*, *GhHCT39*, and *GhHCT61*; *GhHCT8* with *GhHCT40* and *GhHCT62*; *GhHCT9* with *GhHCT20*, *GhHCT41*, and *GhHCT59*). Chromosomes A10 (*GhHCT20* with *GhHCT9* and *GhHCT59*, *GhHCT21* with *GhHCT60*, *GhHCT24* with *GhHCT7*, *GhHCT39*, *GhHCT61*, and *GhHCT26* with *GhHCT62*) and chromosome A08 (*GhHCT17* with *GhHCT19*, *GhHCT27*, *GhHCT57*, *GhHCT63*, and *GhHCT18* with *GhHCT58*) followed. These findings indicate that these chromosomal segments underwent duplication events during evolution, which likely played a significant role in the evolution and expansion of the *GhHCT* gene family.

### 3.7. Expression Analysis of GhHCT Family Genes in Response to V. dahliae Infection in Cotton

**Figure 6:**
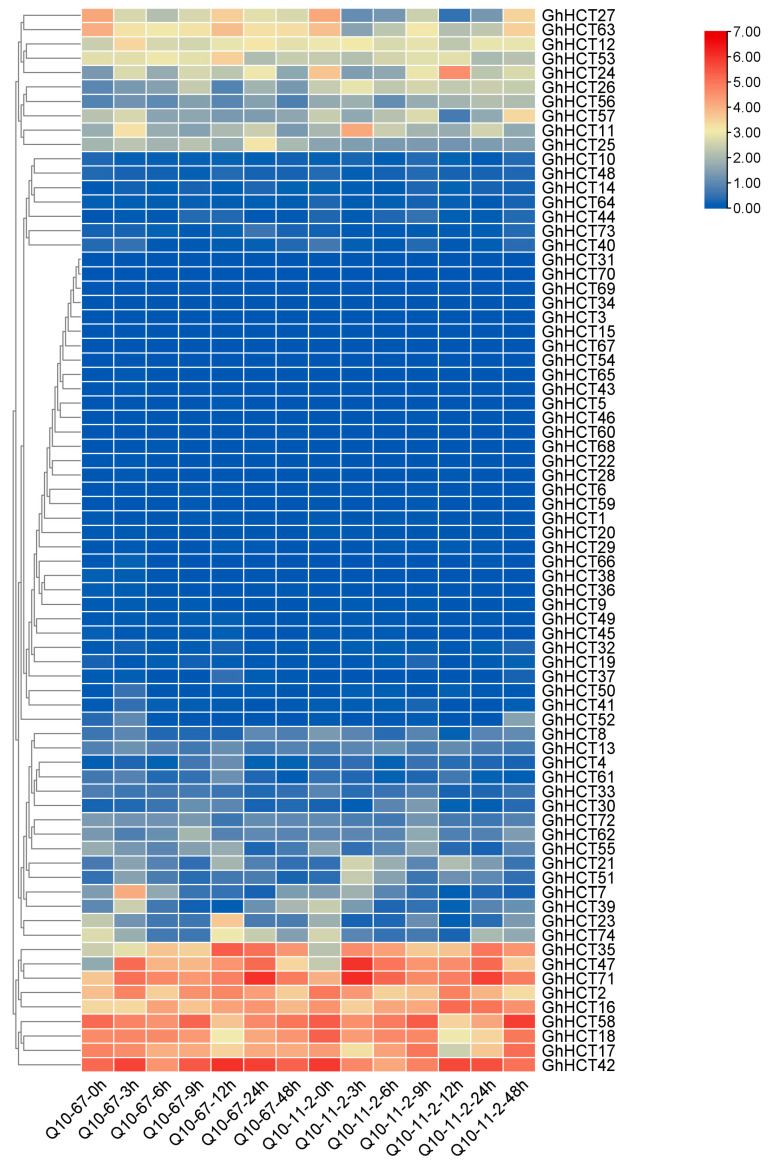
Heatmap of *GhHCT* gene family expression at different time points during *V. dahliae* infection in different cotton varieties. The color gradient represents log_2_ fold change, ranging from higher (red) to lower (blue).

Transcriptome analysis of cotton responses to *V. dahliae* infection (Figure 6) revealed significant differences in the expression of *GhHCT2*, *GhHCT35*, *GhHCT42*, *GhHCT47*, *GhHCT58*, and *GhHCT71*.

**Figure 7:**
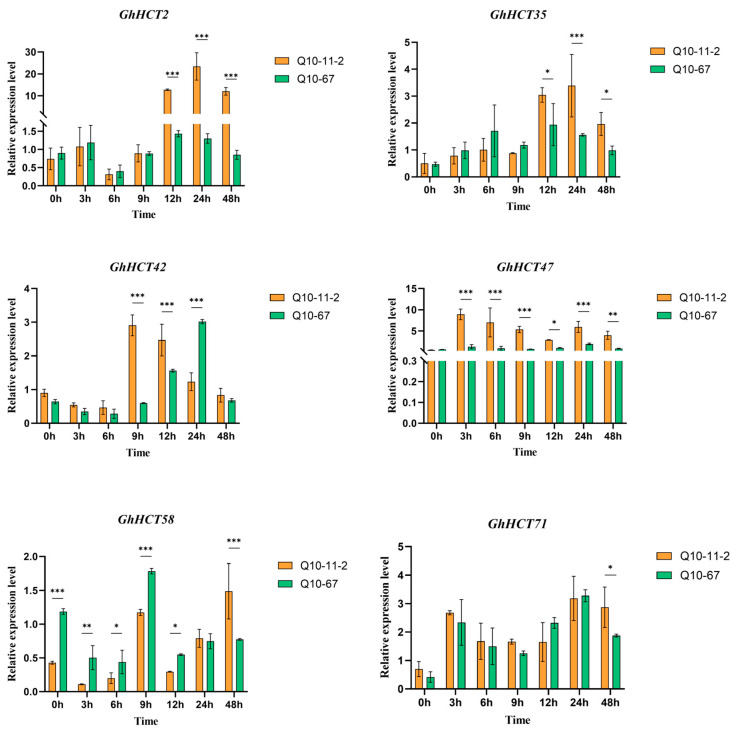
Expression analysis of the key *GhHCT* genes in response to *V. dahliae* treatment in different cotton varieties. qRT-PCR results for 6 differentially expressed *GhHCT* genes at 0 h, 3 h, 6 h, 9 h, 12 h, 24 h, and 48 h post-*V. dahliae* infection. The *GAPDH* gene serves as the internal control. The horizontal axis represents treatment time, and the vertical axis represents average expression levels. Statistical significance is indicated in the figures using asterisks (* *p* < 0.05, ** *p* < 0.01, or *** *p* < 0.001).

Further qRT–PCR analysis of these six *GhHCT* genes (Figure 7) revealed that the expression levels of *GhHCT2*, *GhHCT35*, *GhHCT47* and *GhHCT71* initially increased but then decreased after inoculation with *V. dahliae*. The results showed that at 12 h post-inoculation (hpi), the expression levels of *GhHCT2*, *GhHCT35,* and *GhHCT47* were significantly higher in the resistant cultivar than in the susceptible cultivar (*p* < 0.05). Moreover, the induction fold change in these three genes in the resistant cultivar exceeded that in the susceptible cultivar by more than 2.5-fold at 24 hpi (*p* < 0.01). These statistical comparisons indicate that *GhHCT2*, *GhHCT35*, and *GhHCT47* respond more rapidly and robustly to *V. dahliae* infection in the resistant variety, making them promising candidates for future functional studies aimed at elucidating their roles in early defense mechanisms.

### 3.8. Prediction of the GhHCT Protein–Protein Interaction Network

**Figure 8:**
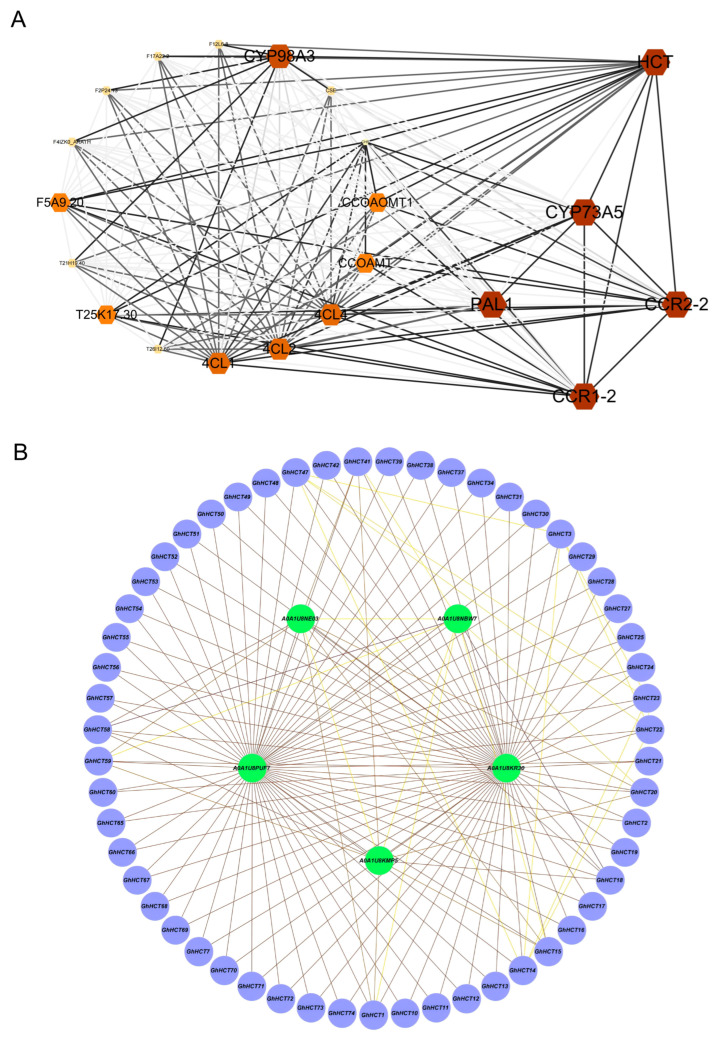
Protein–protein interaction network analysis of *HCT* gene family. (**A**) Inter-family interactions between *HCT* and other gene families; (**B**) intra-family interactions within *HCT* gene family. The intensity of interactions is represented by the circles’ darkness in the figure, with darker circles indicating stronger interactions and lighter circles indicating weaker ones.

To further investigate the function of *GhHCT*, an interaction network analysis of its encoded protein was conducted via the STRING database. As shown in Figure 8, a total of 20 proteins were predicted to potentially interact with HCT, including three 4CL proteins, two CCOAMT proteins, and two CCR proteins. Additionally, PPI prediction revealed that the five members of the *GhHCT* gene family may have potential interactions with each other, and these proteins all belong to the Class IV subfamily (Figure 8B). The abbreviations used in Figure 8 are detailed in Supplementary Table S2.

## 4. Discussion

### 4.1. Identification and Evolutionary Characteristics of the G. hirsutum HCT Gene Family

Hydroxycinnamoyltransferase (HCT) is a key member of the BAHD acyltransferase superfamily, which not only catalyzes chlorogenic acid synthesis but also plays a central role in the phenylpropanoid pathway of lignin biosynthesis [24]. Lignin, a crucial structural component of the cell wall, plays a central role in plant responses to biotic and abiotic stresses [11,28,35]. The functional roles of the *HCT* gene family in lignin synthesis have been studied in plants such as cattail (*Typha angustifolia* L.) [36], *Medicago sativa* L. [37,38], *Vitis vinifera* L. [24], *Gossypium barbadense* [22], and *Camellia sinensis* [39], but its response mechanism to *V. dahliae* in *G. hirsutum* remains unclear.

This study provides the first comprehensive genome-wide analysis of the *HCT* gene family in allotetraploid upland cotton. This study first identified 74 *GhHCT* genes across the *G. hirsutum* genome and classified them into five subfamilies (Classes I–V) based on of phylogenetic relationships and domain characteristics (Figure 4). Protein domain predictions indicate that GhHCT proteins exhibit relatively conserved structures, with Motifs 1, 2, and 3 conserved across all subfamilies (Figure 2), suggesting functional conservation. However, significant variations in gene length and protein domains among subfamilies imply potential for functional diversification.

### 4.2. Regulatory Potential and Expression Specificity of GhHCT Genes

Within intricate regulatory networks, cis-acting regulatory elements serve as central commanders. By precisely orchestrating gene transcription, they govern the orderly progression of numerous biological processes [33,40,41]. This study revealed that the promoter region of the *GhHCT* gene is rich in elements associated with growth and development, hormone responses (including gibberellin, auxin, salicylic acid, and methyl jasmonate), and abiotic stress responses (including low temperature and drought). These findings hint at the possible involvement of the *GhHCT* gene in diverse regulatory networks, including those governing development and stress adaptation. However, as these predictions are based solely on sequence analysis (Figure 3), their functional relevance needs to be empirically tested. Tissue expression pattern analysis provides a theoretical basis for exploring the relationships between gene family members and growth and development [42]. Crucially, multiple *GhHCT* genes were strongly and rapidly induced by *V. dahliae* following inoculation. Among these genes, *GhHCT2*, *GhHCT35*, and *GhHCT47* were significantly upregulated in the resistant cultivar compared to the susceptible one during the early infection phase (3–24 hpi). This expression pattern is consistent with the findings in *A. thaliana*, where HCT expression is rapidly induced upon pathogen challenge and correlates with enhanced disease resistance [22,24]. Similar observations have been reported in grapevine under low-temperature stress and in tea plants in response to biotic stress [24,39], suggesting that HCT-mediated defense responses are evolutionarily conserved across plant species.

### 4.3. Core Role and Mechanism of the GhHCT Gene in Resisting V. dahliae Stress

Lignin is a key structural component of cell walls and plays a central role in plant responses to biotic and abiotic stresses [35]. When plants are subjected to vascular pathogen invasion, complex defense mechanisms are activated. Cell wall lignification is a critical process in which physical barriers are formed to halt pathogen spread within vascular tissues [43,44]. Lignin deposition at infection sites acts as a physical barrier against pathogen invasion [16,18]. More recently, it has been demonstrated that enhanced lignification of vessel walls in cotton restricts the vascular spread of *V. dahliae* [5,18,44,45], highlighting the direct role of lignin in disease resistance. HCT regulates lignin biosynthesis, a finding validated across diverse plant species [2,19,22,23,46]. As a key “traffic controller”, HCT functions through a C3H-dependent double transesterification cycle (p-coumaroyl-CoA⇌p-coumaroylshikimic acid→caffeoylshikimic acid⇌caffeoyl-CoA) [47,48], precisely directing the flow of lignin monomers. The functions of *HCT* genes have been validated across multiple plant species. For example, disrupting its expression in poplar significantly alters lignin content and structure [23]; in *A. thaliana* and *Medicago sativa* L., HCT loss-of-function mutations were shown to reduce lignin content [49,50]. In *A. thaliana* specifically, HCT knockdown lines exhibit reduced lignin content and increased susceptibility to bacterial pathogens [49,51], providing direct evidence for the role of HCT in plant immunity. Collectively, these findings indicate that HCT is evolutionarily conserved in lignin synthesis across diverse plants, positioning it as a critical target for improving crop quality and biomass utilization.

A distinctive contribution of this study lies in its integration of genome-wide identification with cultivar-specific expression profiling under pathogen challenge. In plants, previous studies have confirmed that 4CL enhances resistance to *V. dahliae* by regulating lignin accumulation [52,53]. Functional analysis of the predicted interactors reveals that their corresponding gene families form the core regulatory network of the phenylpropanoid pathway: PAL and C4H initiate the pathway; 4CL determines metabolic branching; and HCT, CCR, and CCOAM synergistically regulate the synthesis of downstream metabolites, such as lignin [54,55]. Based on this network analysis, we hypothesize that *GhHCT* may contribute to plant disease resistance and promote lignin deposition by regulating the phenylpropanoid pathway. This hypothesis can be validated in subsequent studies using techniques such as yeast two-hybrid (Y2H) and Bimolecular Fluorescence Complementation (BiFC) assays. Based on the aforementioned theoretical framework and the expression profiling analysis conducted in this study, we focused on genes that showed sustained high expression following inoculation and were positively correlated with disease resistance. Through comprehensive evaluation of phylogenetic position, expression induction intensity, and promoter element characteristics, we identified three key candidate genes: *GhHCT2*, *GhHCT35,* and *GhHCT47*. The promoter regions of these genes are rich in methyl jasmonate (MeJA) and salicylic acid (SA) response elements—the core mediators of plant disease resistance signaling pathways. Therefore, we hypothesize that *GhHCT2*, *GhHCT35*, and *GhHCT47* could be specifically activated by *V. dahliae* stress through MeJA/SA signaling, thereby enhancing lignification in vascular cells and ultimately limiting further pathogen spread. This hypothesis is consistent with recent studies showing that HCT-mediated lignin deposition contributes to resistance against *V. dahliae* in cotton and other crops [22,39].

In summary, this study represents the first genome-wide systematic analysis of the *HCT* gene family in upland cotton, comprehensively characterizing 74 *GhHCT* genes with respect to evolutionary relationships, structural diversity, and expression patterns. Three key candidate genes—*GhHCT2*, *GhHCT35*, and *GhHCT47*—were identified as specifically and rapidly induced in resistant cultivars upon *V. dahliae* infection, establishing a mechanistic link between HCT-mediated lignin biosynthesis and *V. dahliae* resistance. These findings have significant implications for cotton breeding, as these genes could serve as molecular markers for screening resistant germplasm or as direct targets for genetic improvement via overexpression, genome editing, or marker-assisted selection. Future studies should validate their functions in plants using genetic approaches such as VIGS, CRISPR/Cas9, and overexpression, combined with metabolomics analysis to investigate changes in lignin monomers, thereby fully elucidating the core role of the *HCT* gene family in cotton resistance to *V. dahliae* and providing a solid theoretical basis for disease-resistant breeding.

## 5. Conclusions

This study provides the first comprehensive characterization of the *HCT* gene family in allotetraploid upland cotton (*G. hirsutum*), identifying 74 members at the whole-genome level. These genes are unevenly distributed across the 20 chromosomes of *G. hirsutum*. Proteins within the *GhHCT* gene family exhibit relatively conserved structures, with Motif 1, Motif 2, and Motif 3 serving as conserved motifs shared across all subgroups. Gene structure analysis revealed that most members of the *GhHCT* gene family lack introns. Furthermore, the promoters of *GhHCT* family members contain cis-acting elements associated with plant growth and development, hormone responses, and stress tolerance. Phylogenetic tree and collinearity analyses revealed that 225 *HCT* genes are grouped into five subfamilies. *G. hirsutum* chromosome A05 harbors the greatest number of homologous gene pairs, which may share similar functions. RNA-seq and qRT–PCR analyses, combined with statistical comparison between resistant and susceptible cultivars, revealed that *GhHCT2*, *GhHCT35*, and *GhHCT47* are significantly upregulated upon *V. dahliae* infection, with a faster and stronger response in the resistant genotype. The expression patterns of these genes, together with their promoter characteristics, suggest that they may contribute to defense responses by potentially interacting with defense signaling pathways and phenylpropanoid metabolic networks. However, direct functional validation through genetic and biochemical approaches is required to confirm their proposed roles in promoting lignin deposition and restricting pathogen spread. This study provides a solid foundation for further elucidating the functions of *GhHCT* genes and disease resistance mechanisms in cotton.

## Figures and Tables

**Table 1:** Physical and chemical properties of the *GhHCT* gene family in *G. hirsutum*.

Gene Rename	Gene Name	Protein Length (aa)	Molecular Weight (KDa)	Isoelectric Point	Instability Index	Aliphatic Index	GRAVY	Subcellular Localization Predicted
*GhHCT1*	*GhChrA03G0712.1*	433	47.84	5.25	32.12	95.03	−0.048	Cytoplasm
*GhHCT2*	*GhChrA04G1205.1*	466	51.40	8.24	33.8	81.14	−0.195	Chloroplast
*GhHCT3*	*GhChrA05G0578.1*	433	48.25	7.16	44.15	85.59	−0.202	Cytoplasm
*GhHCT4*	*GhChrA05G1241.1*	457	50.90	5.4	49.48	90	−0.053	Cytoplasm
*GhHCT5*	*GhChrA05G1244.1*	461	51.41	5.32	50.6	88.35	−0.059	Cytoplasm
*GhHCT6*	*GhChrA05G1245.1*	456	50.56	6.04	49.65	88.27	−0.118	Cytoplasm
*GhHCT7*	*GhChrA05G3785.1*	477	53.89	8.59	38.97	79.94	−0.223	Nucleus
*GhHCT8*	*GhChrA05G3821.1*	442	48.42	8.68	31.68	90.41	−0.038	Cytoplasm
*GhHCT9*	*GhChrA05G3901.1*	445	49.10	5.64	35.34	92.22	−0.073	Cytoplasm
*GhHCT10*	*GhChrA06G0077.1*	448	50.66	6.95	38.55	96.76	−0.085	Cytoplasm
*GhHCT11*	*GhChrA06G0199.1*	455	50.77	6.02	42.54	86.77	−0.149	Nucleus
*GhHCT12*	*GhChrA06G0200.1*	455	50.98	5.89	43.06	88.04	−0.161	Nucleus
*GhHCT13*	*GhChrA06G0589.1*	458	50.90	7.57	45.84	87.1	−0.007	Nucleus
*GhHCT14*	*GhChrA06G2600.1*	445	50.18	9.06	37.32	81.75	−0.346	Nucleus
*GhHCT15*	*GhChrA07G0597.1*	438	48.76	5.48	31.59	100.16	0.019	Cytoplasm
*GhHCT16*	*GhChrA07G2867.1*	478	53.33	8.03	37.08	85.04	−0.118	Cytoplasm/Nucleus
*GhHCT17*	*GhChrA08G1431.1*	441	48.94	6.2	34.64	81.79	−0.284	Cytoplasm
*GhHCT18*	*GhChrA08G3015.1*	449	50.09	5.54	35.62	88.82	−0.106	Cytoplasm
*GhHCT19*	*GhChrA09G0511.1*	445	49.60	6	35.38	80.45	−0.309	Cytoplasm
*GhHCT20*	*GhChrA10G0715.1*	443	48.92	5.59	30.34	90.27	−0.112	Cytoplasm
*GhHCT21*	*GhChrA10G1754.1*	456	51.50	7.28	43.55	84.01	−0.177	Nucleus
*GhHCT22*	*GhChrA10G1819.1*	436	48.66	7.7	34.46	85.21	−0.263	Cytoplasm
*GhHCT23*	*GhChrA10G1836.1*	436	48.55	6.03	34.35	95.05	−0.147	Cytoskeleton
*GhHCT24*	*GhChrA10G2916.1*	480	53.84	8.23	42.96	73.33	−0.247	Nucleus
*GhHCT25*	*GhChrA10G2917.1*	466	52.15	8.57	44.06	74.1	−0.266	Chloroplast
*GhHCT26*	*GhChrA10G2987.1*	444	48.91	9.6	31.68	93.04	−0.012	Cytoplasm
*GhHCT27*	*GhChrA11G0125.1*	451	49.64	5.48	32.05	78.23	−0.31	Cytoskeleton
*GhHCT28*	*GhChrA11G0828.1*	464	51.45	7.16	30.67	86.81	−0.124	Cytoplasm
*GhHCT29*	*GhChrA11G2229.1*	440	49.08	6.35	33.4	88.23	−0.064	Chloroplast
*GhHCT30*	*GhChrA11G2230.1*	440	48.76	6.64	31.28	84.68	−0.177	Chloroplast
*GhHCT31*	*GhChrA11G2231.1*	433	48.88	5.83	38.83	92.56	−0.153	Mitochondrion
*GhHCT32*	*GhChrA12G1811.1*	496	54.97	6.5	40.53	82.94	−0.047	Chloroplast
*GhHCT33*	*GhChrA13G0475.1*	470	52.26	6.15	43.6	86.62	−0.029	Cytoplasm
*GhHCT34*	*GhChrA13G1398.1*	448	49.99	6.32	40.03	91.65	−0.113	Cytoplasm
*GhHCT35*	*GhChrA13G2137.1*	454	50.44	7.57	41.77	91.06	−0.027	Cytoplasm
*GhHCT36*	*GhChrA13G2139.1*	435	48.41	6.39	36.18	98.67	0.139	Cytoplasm
*GhHCT37*	*GhChrA13G2140.1*	461	51.23	7.94	43.2	93.73	0.041	Chloroplast
*GhHCT38*	*GhChrA13G2141.1*	435	48.51	6.39	38.65	99.1	0.12	Cytoplasm
*GhHCT39*	*GhChrD04G0770.1*	477	53.69	8.2	39.33	79.52	−0.232	Cytoplasm
*GhHCT40*	*GhChrD04G0805.1*	442	48.29	8.67	31.83	91.09	−0.028	Cytoplasm
*GhHCT41*	*GhChrD04G0868.1*	445	49.15	5.64	35.8	91.35	−0.086	Cytoplasm
*GhHCT42*	*GhChrD04G1682.1*	466	51.47	7.97	36.15	81.97	−0.182	Chloroplast
*GhHCT43*	*GhChrD05G0573.1*	433	48.36	7.62	42.15	86.26	−0.203	Cytoplasm
*GhHCT44*	*GhChrD05G1224.1*	451	50.32	5.37	50.09	88.82	−0.035	Cytoplasm
*GhHCT45*	*GhChrD05G1225.1*	461	51.39	5.53	50.46	90.04	−0.057	Cytoplasm
*GhHCT46*	*GhChrD05G1226.1*	452	50.35	5.93	51.14	86.24	−0.131	Cytoplasm
*GhHCT47*	*GhChrD05G1240.1*	467	52.02	6.47	34.73	82.06	−0.239	Mitochondrion
*GhHCT48*	*GhChrD06G0074.1*	448	50.61	6.59	38.79	95.69	−0.09	Nucleus
*GhHCT49*	*GhChrD06G0183.1*	454	50.64	6.28	48.18	87.58	−0.069	Cytoplasm
*GhHCT50*	*GhChrD06G0188.1*	455	51.18	5.93	49.65	86.53	−0.145	Cytoplasm
*GhHCT51*	*GhChrD06G0195.1*	455	50.65	6.32	42.41	86.13	−0.16	Cytoplasm
*GhHCT52*	*GhChrD06G0321.1*	438	48.82	5.84	35.06	92.79	−0.062	Cytoplasm
*GhHCT53*	*GhChrD06G0565.1*	457	50.76	6.79	46.49	83.22	−0.014	Nucleus
*GhHCT54*	*GhChrD06G0566.1*	473	52.99	6.07	45.16	83.04	−0.049	Cytoplasm
*GhHCT55*	*GhChrD07G2756.1*	472	52.33	6.1	34.88	83.03	−0.136	Nucleus
*GhHCT56*	*GhChrD07G2757.1*	471	52.10	5.97	36.19	86.73	−0.136	Chloroplast
*GhHCT57*	*GhChrD08G1324.1*	441	48.98	5.8	34.96	80.68	−0.317	Cytoplasm
*GhHCT58*	*GhChrD08G2898.1*	449	49.79	5.54	33.93	89.47	−0.072	Cytoplasm
*GhHCT59*	*GhChrD10G0858.1*	443	48.99	5.93	30.13	90.93	−0.108	Cytoplasm
*GhHCT60*	*GhChrD10G1381.1*	457	51.59	8.61	43.89	84.68	−0.177	Nucleus
*GhHCT61*	*GhChrD10G2819.1*	480	53.86	8.38	41.25	74.75	−0.247	Chloroplast
*GhHCT62*	*GhChrD10G2879.1*	447	49.38	9.41	32.05	93.71	−0.007	Cytoplasm
*GhHCT63*	*GhChrD11G0113.1*	473	52.41	5.57	30.07	82.22	−0.238	Chloroplast
*GhHCT64*	*GhChrD11G0825.1*	464	51.38	6.47	31.4	88.69	−0.081	Cytoplasm
*GhHCT65*	*GhChrD11G2217.1*	433	48.93	5.93	40.93	93.9	−0.144	Mitochondrion

## Data Availability

The data presented in this study may be obtained from the corresponding author or first author upon request.

## Supplementary Materials

The following supporting information can be downloaded at: https://www.mdpi.com/article/10.3390/biology15070520/s1.
