# Supplementary material for: Genome-Wide Identification and Expression Analysis of the HCT Gene Family in Upland Cotton (Gossypium hirsutum L.) in Response to Verticillium wilt Infection
Source: Biology (Basel). 2026 Mar 25;15(7):520. doi: 10.3390/biology15070520 (PMC13072020; doi:10.3390/biology15070520)
Supplement: Supplementary file 1 [file biology-15-00520-s001.zip › Supplementary Document S1.pdf]

Specifically, we have added the following information:

Library Construction and Sequencing (Platform and Depth): We have detailed the entire process from RNA extraction to sequencing. Total RNA was extracted using a polysaccharide/polyphenol-rich plant RNA extraction kit. Sequencing libraries were constructed following the manufacturer's instructions using an RNA library preparation kit. The libraries were quality-controlled via Qubit 2.0 for initial quantification and Agilent 2100 for insert size detection, followed by precise quantification using Q-PCR (2 nM). The qualified libraries were pooled and sequenced on the Illumina NovaSeq X Plus platform with PE150 (paired-end 150 bp) mode. A total of 358.75 Gb of raw data was generated from 42 samples, with each sample yielding at least 7.50 Gb of Clean Data, and the Q30 percentage was above 97.61%.

Bioinformatic Pipeline (Quality Control and Mapping): We have clarified the software and parameters used. Fastp (v0.20.0) was employed to filter raw reads, removing adapter sequences and low-quality reads to obtain Clean Data. The clean reads were then aligned to the reference genome (TM-1-T2T) using HISAT2 (v2.1.0) to generate mapped data.

Differential Expression Analysis (Thresholds): For the identification of differentially expressed genes (DEGs) across different time points after Verticillium dahliae infection, we utilized DESeq2. Genes with an absolute $|Log_2(\text{Fold Change})| \geq 1$ and a False Discovery Rate (FDR) < 0.05 were considered significantly differentially expressed.
