# Supplementary figures and images for: Genome-Wide Identification and Expression Analysis of the HCT Gene Family in Upland Cotton (Gossypium hirsutum L.) in Response to Verticillium wilt Infection
Source: Biology (Basel). 2026 Mar 25;15(7):520. doi: 10.3390/biology15070520 (PMC13072020; doi:10.3390/biology15070520)

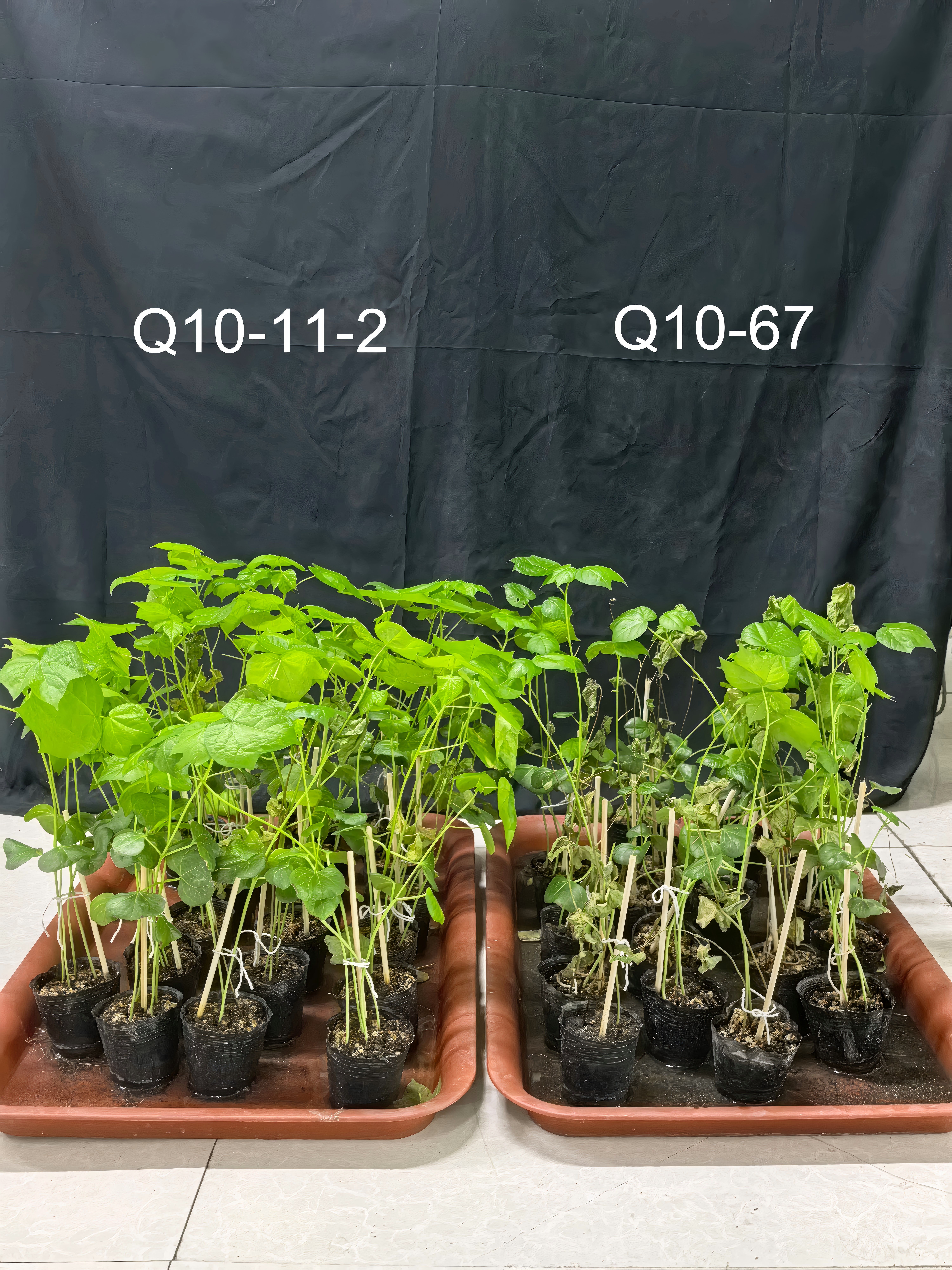

Supplement: Supplementary file 1 [file biology-15-00520-s001.zip › Supplementary Figure S1.png]
